# Correction to “The relationship between interhemispheric synchrony, morphine and microstructural development of the corpus callosum in extremely preterm infants”

**DOI:** 10.1002/hbm.26637

**Published:** 2024-03-15

Failla, A., Filatovaite, L., Wang, X., Vanhatalo, S., Dudink, J., de Vries, L. S., Benders, M., Stevenson, N., & Tataranno, M. L. (2022). The relationship between interhemispheric synchrony, morphine and microstructural development of the corpus callosum in extremely preterm infants. Human Brain Mapping, 43(16), 4914–4923. https://doi.org/10.1002/hbm.26040

In Table 3 of the “Results” section, the *b* coefficients and *p* values were incorrect. This should have been the following:

**TABLE 3:** ASI and morphine until the first week of life, as either dosage or yes/no‐measurement.

	ASI (as AUC until W1; *n* = 25)	
	*b* (CI 95%) [*p* value]	
Morphine dosage until W1	‐	8.061 (2.926; 13.195) [.004**]
Morphine Yes/No until W1	9.960 (2.545; 17.374) [.011*]	‐
BW *z*‐score	*−1.202 (−4.565; 2.162)* [.466]	−1.719 (−4.936; 1.497) [.280]

*Note*: Rows are showing morphine and birth weight included in the multivariable linear regression analysis. Column shows ASI value as a single value until the first week of life. The *b* values with CI and *p* values are shown. *indicates *p* value <.05 and **indicates a *p* value <.01. Abbreviations: ASI, activation synchrony index; BW, birth weight; CI, confidence intervals.

We apologize for this error.

